# Peri-implant assessment via cone beam computed tomography and digital periapical radiography: an *ex vivo* study

**DOI:** 10.6061/clinics/2017(11)10

**Published:** 2017-11

**Authors:** Nicolau Silveira-Neto, Mateus Ericson Flores, João Paulo De Carli, Max Dória Costa, Felipe de Souza Matos, Luiz Renato Paranhos, Maria Salete Sandini Linden

**Affiliations:** I Departamento de Odontologia, Universidade de Passo Fundo, Passo Fundo, RS, BR; II Departamento de Odontologia, Universidade Tiradentes, Aracaju, SE, BR; III Departamento de Odontologia Restauradora, Instituto de Ciência e Tecnologia, Universidade Estadual Paulista, São José dos Campos, SP, BR; IV Departamento de Odontologia, Universidade Federal de Sergipe, Lagarto, SE, BR

**Keywords:** Artifacts, Cone Beam Computed Tomography, Dental Implants

## Abstract

**OBJECTIVES:**

This research evaluated detail registration in peri-implant bone using two different cone beam computer tomography systems and a digital periapical radiograph.

**METHODS:**

Three different image acquisition protocols were established for each cone beam computer tomography apparatus, and three clinical situations were simulated in an *ex vivo* fresh pig mandible: buccal bone defect, peri-implant bone defect, and bone contact. Data were subjected to two analyses: quantitative and qualitative. The quantitative analyses involved a comparison of real specimen measures using a digital caliper in three regions of the preserved buccal bone – A, B and E (control group) – to cone beam computer tomography images obtained with different protocols (kp1, kp2, kp3, ip1, ip2, and ip3). In the qualitative analyses, the ability to register peri-implant details via tomography and digital periapical radiography was verified, as indicated by twelve evaluators. Data were analyzed with ANOVA and Tukey’s test (α=0.05).

**RESULTS:**

The quantitative assessment showed means statistically equal to those of the control group under the following conditions: buccal bone defect B and E with kp1 and ip1, peri-implant bone defect E with kp2 and kp3, and bone contact A with kp1, kp2, kp3, and ip2. Qualitatively, only bone contacts were significantly different among the assessments, and the p3 results differed from the p1 and p2 results. The other results were statistically equivalent.

**CONCLUSIONS:**

The registration of peri-implant details was influenced by the image acquisition protocol, although metal artifacts were produced in all situations. The evaluators preferred the Kodak 9000 3D cone beam computer tomography in most cases. The evaluators identified buccal bone defects better with cone beam computer tomography and identified peri-implant bone defects better with digital periapical radiography.

## INTRODUCTION

The success of rehabilitation with dental implants requires adequate preoperative planning. Imaging diagnosis and planning methods frequently target the assessment of sites proposed for implant bonding, including the analysis of two-dimensional conventional radiographs such as periapical, interproximal, and panoramic radiographs 1-3. The greatest limitation of these methods is the inherent magnification, distortion and lack of three-dimensional information 4.

In recent years, cone beam computed tomography (CBCT) has become an important alternative diagnostic tool with high potential for diagnosis and treatment planning, especially for implant treatment 5, by providing three-dimensional images 6,7, which are desirable for determining buccolingual thickness and the morphology and inclination of the alveolar bone 1,4,8. However, if metal, for example, in metal restorations or dental implants, is present in the tomography area, the images tend to display artifacts, which are the main cause of decreased image quality, rendering the images useless for diagnosis in some cases 9,10.

Artifacts produced by metal structures, such as titanium dental implants, represent a challenge for automatic processing using computed tomography (CT) scanner software 5,9. Compared to bone or soft tissue, metallic implants lead to considerable X-ray beam attenuation. This attenuation may produce shadows or beam hardening, which are often obstacles for detailing structures close to these metals, and complicates the postoperative assessment of dental implants with CT 9,11-13.

This study aimed to verify detail registration in the peri-implant region using CBCT and digital periapical radiograph exams. Three postoperative situations were simulated, and three different image acquisition protocols were used for two CBCT scanners, considering the uncertainty regarding the optimal protocol for tomographic image acquisitions with respect to the reduction of metal artifacts and higher registration accuracy in clinical situations.

## MATERIALS AND METHODS

### Production of an Experimental Model

In a fresh pig mandible (*ex vivo*), an installation was planned with three cylindrical, external hexagonal titanium dental implants that were 3.75 x 15 mm in size (Conexão Sistemas de Prótese, São Paulo, SP, Brazil) and spaced at least 5 mm apart using a surgical guide previously made with a casting and mandible plaster model.

The perforations for implant installations simulated an *in vivo* installation according to the manufacturer’s instructions for working with medullary bone, with purposeful under instrumentation to produce the highest bone contact with the implants. A motor (Driller BLM 600 Plus, Driller, São Paulo, SP, Brazil) and a 20:1 surgical contra-angle handpiece (W&H Dentalmechanik, Bürmos, Austria) were used at 1000 rpm under constant saline solution irrigation. The burs used were P-i Branemark™ diamond-like carbon (Exopro, São Paulo, SP, Brazil).

**Figure 1:**
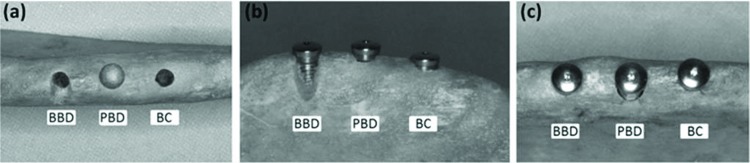
(a) Upper view of the planned clinical situations: BBD, PBD, and BC. (b) and (c) Buccal and upper views of the installed implants showing the three clinical situations.

Before implant installation, the cervical regions were assessed to produce three clinical situations (Figure 1): buccal bone defect (BBD), which was simulated by buccal wear with a KG Sorensen 3215 diamond bur (KG Sorensen, Cotia, SP, Brazil) under high rotation using a Kavo Extra Torque 650C (Kavo, Joinville, SC, Brazil); peri-implant bone defect (PBD), which was simulated by enlargement of the surgical cavity with a 5-mm-diameter Dense Drill from P-i Branemark™ (Exopro, São Paulo, SP, Brazil), preserving 4 mm of bone contact in the apical region for implant stability at the moment of installation; and bone contact (BC), which was a full bone overlay with no bone defect simulation.

Next, implants were installed according to the manufacturer’s instructions. Initially, an internal grip driver from P-i Branemark™ (Exopro, São Paulo, SP, Brazil) was used with a motor at 26 rpm, and a manual torque wrench from P-i Branemark™ (Exopro, São Paulo, SP, Brazil) was used to complete the insertion.

### Image Acquisition

To test the hypothesis that detail registration in peri-implant regions is influenced by the image acquisition protocol used, two CBCT scanners were used: a Kodak 9000 3D CBCT (Dental Systems, Carestream Health, Rochester, USA) and an i-CAT CBCT (Imaging Sciences International, Hatfield, USA). Three image acquisition protocols were used for each apparatus (Tables 1 and 2).

**Figure 2:**
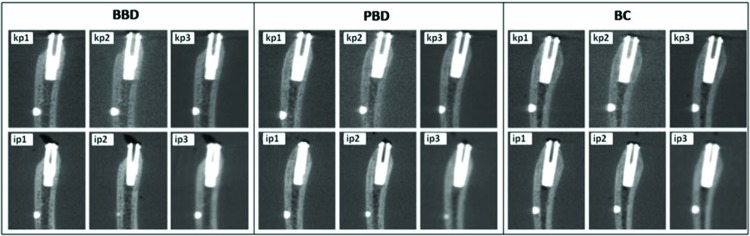
Image results from the BBD, PBD, and BC cases for the different image acquisition protocols from sagittal CBCT images.

A container with the experimental model was placed on the exam platform for each CT scanner and was fixed and aligned using guidance lasers. After CT exams, the software of each scanner standardized the transverse oblique-sectioned images and the most central specimen section of each implant. The images were organized in three templates corresponding to each postoperative situation (BBD, PBD, and BC) (Figure 2).

A periapical radiograph was also taken using a charge-coupled device (CCD) digital sensor of the Kodak RVG 6100 (Kodak Dental System) model. Each implant was radiographed at an orthoradial angle with the parallelism technique following the geometric principles of radiographic image formation.

After image acquisition, the specimen was sectioned in a cutting machine (Miniton, Struers, Copenhagen, Denmark) with a diamond disc at the central point of the implants and parallel to the long axis in order to match the images previously selected in the CT scanner software. After sectioning, the portions chosen for implant assessment were photographed with a digital camera (Nikon D80, Nikon, Japan) using a macro lens and a Sigma circular flash for Nikon (Sigma, Japan) for qualitative analysis.

**Figure 3:**
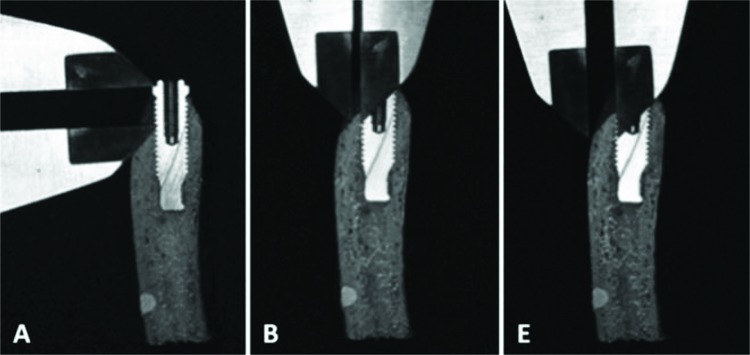
Measurements of a real object made with a digital caliper in regions A, B, and E.

Moreover, a properly calibrated examiner measured the real object using a digital caliper (Cosa Vinhedo, SP, Brazil) in three regions of the preserved buccal portion (Figure 3): region A corresponded to the vertical measure (height) of the prosthetic platform of the implant up to the first buccal bone contact to the implant, region B was the bone thickness measure of the first buccal bone contact to the implant, and region E indicated the buccal bone thickness measure in the middle third of the implant.

These measurements were repeated five times for each region at different times, and the following control (CG) measures were considered: CBBD-A, CPBD-A, and CBC-A - vertical buccal measures for region A in each implant, CBBD-B, CPBD-B, and CBC-B - horizontal buccal measures for region B in each implant, CBBD-E, CPBD-E, and CBC-E - horizontal buccal measures for region E in each implant.

The same measurements were also taken in these regions using the images obtained from each CT scanner software program with the different image acquisition protocols. The images were assessed by two experienced and trained dentists for each scanner, one dentist for each scanner studied. Three measurements were taken at different times for each region.

### Data Analysis

All quantitative data were tabulated and analyzed with ANOVA and Tukey’s test (α=0.05). Qualitative assessment was performed by twelve dentists experienced in implantology or dental radiology and in the routine use of CT images in order to verify which CT scanners and acquisition protocols were able to more accurately register the study object. The dentists performed an isolated and blind assessment, as they were not informed as to which CT scanner produced the image shown.

**Figure 4:**
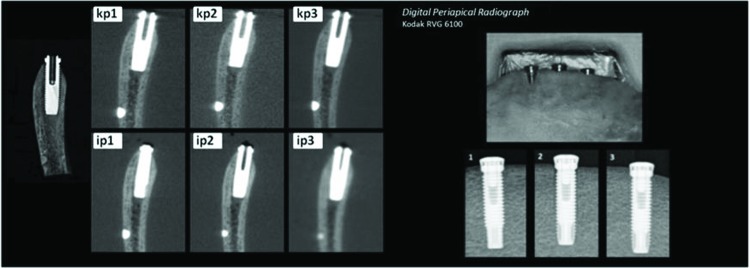
Model of presentation for evaluators for the PBD situation.

During the assessment, the evaluator was presented with a photograph of the transverse oblique section of the real object, images corresponding to each CT scanner and protocol used, a photograph of the buccal aspect of the specimen before sectioning, and periapical radiographs of each implant (Figure 4).

Each dentist was asked to rate the ability of the tomographic image to accurately register peri-implant details in each situation on a scale 14 from 1 to 5, with the following score options: 1 - very poor, 2 - poor, 3 - good, 4 - very good, 5 - excellent. The data were analyzed with ANOVA and Tukey’s test (α=0.05).

## RESULTS

### Quantitative Analysis

Tables 3 and 4 present the mean values for the different image acquisition protocols (Kodak - kp1, kp2, kp3 and i-CAT - ip1, ip2, ip3) as well as the true values found by direct measurement (CG) in the three regions measured (region A, B, and E) for the three simulated situations (BBD, PBD, and BC).

### Qualitative Analysis

According to the scores assigned by the evaluators, similar performances were verified for the images from both CT scanners; only the BC situation gave significantly different results. The results for acquisition protocol 3 differed from those for 1 and 2. The results for the other protocols were not significantly different (Table 5). The interpretation of scores for tomographic images and digital periapical radiographs in each situation in terms of the ability of each image to register the simulated bone condition is shown in Table 5.

## DISCUSSION

CT is an important imaging method for the diagnosis of lesions in both hard and soft tissues of the oral cavity and the head and neck region. In recent years, CBCT has become an important alternative diagnostic tool due to its high potential for diagnosis and treatment planning, especially for implant treatment, by providing three-dimensional images 1,5-8.

However, artifacts cause decreased image quality in CBCT, which in some cases may render such images useless for diagnosis 9,10. An artifact is any type of image distortion or error that is not related to the object of study. Artifacts produced by metal structures, such as titanium dental implants, represent a challenge for automatic processing using CT scanner software 5,9. Schulze et al 15 assessed the image quality of the New Tom 900 and Siemens Siremobil in a dehydrated skull and reported no artifact formation, which was attributed to the fact that no metal structures were used in the study.

The present work observed different quantities of metal artifacts in all exams due to the presence of titanium implants; compared to bone or soft tissue, titanium implants cause considerable X-ray beam attenuation. This attenuation may produce shadows or beam hardening, which are often obstacles for detailing structures close to these metals, complicating the postoperative assessment of dental implants with CT 9-13.

The use of higher kVp (kp3 - Kodak 9000 3D CBCT) and mAs (ip1 - i-CAT CBCT) values allowed for images with measures statistically equal to those of the CG only in the kp3-PBD region E, kp3-BC region A, and ip1-BBD region B situations. Kataoka et al 11 suggested that an increase in kVp and mAs may help control metal artifact formation. Esmaeili et al 10 also reported that the high resolution of images produced by the New Tom VGi and Somatom Sensation may be attributed to the high kVp used for image acquisition. Corroborating these authors, Chindasombatjareon et al 6, when assessing the production of metal artifacts in the Light Speed QX/I CBCT and Alpha Veja 3030 CBCT systems, observed that an increase in kVp in both CT scanners led to a reduction in artifacts.

In contrast, the use of lower kVp (kp1 - Kodak 9000 3D CBCT) and mAs (ip3 - i-CAT CBCT) values also resulted in images with measures statistically equal to the those of the CG in the kp1-BBD region B, kp1-BBD region E, and kp1-BC region A situations. Shulze et al 13, in a study performed using a phantom with CBCT, found similar results and affirmed that using a lower kVp (80 compared to 90 and 120) resulted in reduced beam hardening.

Some intermediate values of kVp showed irregularities in results when the voxel size (kp2 - Kodak 9000 3D CBCT) and mAs (ip2 - i-CAT CBCT) were reduced, producing images with measures statistically equal to those of the CG in the kp2-PBD region E, kp2-BC region A, and ip2-BC region A situations. Similarly, Ravazi et al 12, in a study performed on a bovine rib with i-CAT NG (120 kV, 18.54 mAs, voxel 0.3, FOV 8x16 cm) and Accuitomo 3D60 FPD (80 kVp, 4 mAs, voxel 0.125 mm, FOV 6x6 cm), verified that both instruments overestimated the distance from the implant vertical to the bone crest, with better images obtained by the Accuitomo 3D60 FPD. The present study also showed that the ip3 acquisition protocol had the most disparate results from the CG. This result may be explained by the larger voxel size and lower mAs among the protocols used for i-CAT CBCT.

All protocols used for the Kodak 9000 3D CBCT had lower kVp and mAs values than those for the i-CAT CBCT. There was a variation between underestimation and overestimation of screw display and bone thickness analyzed, with some cases suggesting a higher bone loss than that found in the real object. Other cases suggested the presence of bone tissue in places not found in the real object. The kp1-BBD region A, kp2-BBD region A, and kp3-BBD region A situations showed higher average measures than those of the CG. In contrast, the protocols set for i-CAT had an overall tendency to reduce the average measures relative to the CG.

The qualitative assessment indicated a superiority of the Kodak 9000 3D CBCT for BBD and BC situations because, according to the evaluators, artifact formation did not compromise the identification of peri-implant characteristics in these situations. For the PBD situation, both CBCT apparatuses obtained intermediate scores, with i-CAT obtaining a higher score, which may be attributed to the use of higher kVp and mAs values in the exam acquisitions, preventing masking of the bone defect simulated around the implant by artifacts. Statistically, according to the scores assigned by the evaluators, only the BC situation produced different results, where the p3 results were different from the p1 and p2 results.

The dentists involved in the qualitative assessment were surprised, especially when observing the images simulated for the PBD case, that no acquisition protocol could reduce the formation of artifacts and accurately portray the real condition, which suggests, for some situations examined such as kp1, kp2, kp3, and ip3, a completely non-existent bone contact. The difficulty in defining the region of bone contact with the implants was unanimous for both radiologists and implantologists, which agrees with reports by Ravazi et al 12 and Schulze et al 13.

Some studies have investigated the difference in diagnostic accuracy between CBCT and periapical radiography 1-3,8,16. The majority revealed the superiority of CBCT to periapical radiography for detecting apical lesions. A similar result was found by Estrela et al 16, who found 100% sensitivity in detecting lesions with CBCT and a much lower sensitivity with panoramic (28%) and periapical (55%) radiography. In the present research, the digital periapical radiograph was preferred among evaluators for detecting the simulated PBD, which may be attributed to the absence of artifacts. Due to structural overlaps, which are characteristic of periapical radiographs, the evaluators identified the BBD case better using CBCT.

Considering the relevance of the present study in clarifying diagnostic imaging methods in patients with dental implants, it should be highlighted that the experiment was performed in an *ex vivo* model of a dry swine jaw. This approach differs from the methodology used in previous studies 17,18, presenting a limitation of the present study due to the absence of soft tissue during image acquisition.

The registration of peri-implant details is influenced by the image acquisition protocol, although metal artifacts were produced in all situations. The Kodak 9000 3D CBCT registered the study object more accurately than the i-CAT CBCT. The evaluators preferred digital periapical radiography for detecting the simulated peri-implantitis, while the buccal bone defect was identified better using CBCT.

## AUTHOR CONTRIBUTIONS

Flores ME and Silveira-Neto N were responsible for the conception and design of the study. Flores ME and Linden MS were responsible for project coordination. Matos FS and Paranhos LR drafted the manuscript. Flores ME and Silveira-Neto N were responsible for the data collection. Carli JP and Paranhos LR were responsible for the statistical analyses. Silveira-Neto N, Paranhos LR, Matos FS, Costa MD, Flores ME, Carli JP and Linden MS edited and revised the manuscript.

## Figures and Tables

**Table 1:** Specifications for the Kodak 9000 3D CBCT.

Parameters	kp1	kp2	kp3
kVp	68	60	80
mAs	8.0	6.3	12.0
Voxel	0.076	0.1	0.2
FOV (mm)	4.7 x 5.9	4.7 x 5.9	4.7 x 5.9
Exam time (s)	10.8	10.8	10.8
Software	Kodak Dental Imaging Software 3D	Kodak Dental Imaging Software 3D	Kodak Dental Imaging Software 3D

**Table 2:** Specifications for the i-CAT CBCT.

Parameters	ip1	ip2	ip3
kVp	120	120	120
mAs	37.07	20.27	18.54
Voxel	0.2	0.125	0.4
FOV (mm)	8.0 x 8.0	8.0 x 8.0	8.0 x 8.0
Exam time (s)	26.09	26.09	8.9
Software	i-CAT Vision Xoran	i-CAT Vision Xoran	i-CAT Vision Xoran

**Table 3:** Mean values for the BBD, PBD, and BC cases using the Kodak 9000 3D CBCT.

Protocol	Region A (mm)	Region B (mm)	Region E (mm)
BBD - buccal bone defect			
Control	4.622^b^	0.688^a^	2.750^a^
kp1	4.730^a^	0.704^a^	2.740^a^
kp2	4.686^a^	0.528^b^	2.640^b^
kp3	4.690^a^	0.534^b^	2.604^b^
PBD - peri-implant bone defect			
Control	9.914^a^	3.216^a^	2.858^a^
kp1	8.980^c^	2.856^c^	2.784^b^
kp2	9.676^b^	3.040^b^	2.846^a^
kp3	9.560^b^	2.914^c^	2.810^ab^
BC - bone contact			
Control	0.878^a^	0.862^a^	2.518^a^
kp1	0.914^a^	0.700^b^	2.450^b^
kp2	0.918^a^	0.574^c^	2.408^bc^
kp3	0.872^a^	0.588^c^	2.370^c^

Means followed by the same letter are not significantly different (Tukey’s test, α=0.05).

**Table 4:** Mean values for the BBD, PBD, and BC cases using the i-CAT CBCT.

Protocol	Region A (mm)	Region B (mm)	Region E (mm)
BBD - buccal bone defect			
Control	4.622^a^	0.688^a^	2.750^a^
ip1	4.234^d^	0.620^ab^	2.260^c^
ip2	4.500^b^	0.570^b^	2.510^b^
ip3	4.384^c^	0.394^c^	2.020^d^
PBD - peri-implant bone defect			
Control	9.914^a^	3.216^a^	2.858^a^
ip1	8.712^c^	2.592^b^	2.632^b^
ip2	8.980^b^	2.586^b^	2.510^c^
ip3	8.888^bc^	2.460^c^	2.010^d^
BC - bone contact			
Control	0.878^a^	0.862^a^	2.518^a^
ip1	0.648^c^	0.534^c^	2.388^b^
ip2	0.856^a^	0.564^c^	2.250^c^
ip3	0.764^b^	0.724^b^	1.736^d^

Means followed by the same letter are not significantly different (Tukey’s test, α=0.05).

**Table 5:** Score interpretations for tomographic images and digital periapical radiographs in each situation.

Situation	Kodak	i-CAT	Digital Periapical Radiograph
BBD	p1 - Very Good	p1- Good	Very Poor
	p2 - Very Good	p2 - Good	
	p3 - Very Good	p3 - Good	
PBD	p1 - Very Poor	p1 - Poor	Very Good
	p2 - Very Poor	p2 - Very Poor	
	p3- Very Poor	p3 - Very Poor	
BC	p1- Very Good	p1 - Poor	Very Good
	p2 - Very Good	p2 - Good	
	p3- Poor	p3 - Poor	
